# Pediatric medulloblastoma express immune checkpoint B7-H3

**DOI:** 10.1007/s12094-021-02762-y

**Published:** 2022-01-05

**Authors:** S. Li, G. C. Poolen, L. C. van Vliet, J. G. Schipper, R. Broekhuizen, M. Monnikhof, W. Van Hecke, J. F. Vermeulen, N. Bovenschen

**Affiliations:** 1 Department of Pathology, University Medical Center Utrecht, Heidelberglaan 100, 3584 CX Utrecht, The Netherlands; 2 Center for Translational Immunology, University Medical Center Utrecht, 3584 CX Utrecht, The Netherlands

**Keywords:** Pediatric medulloblastoma, Brain cancer, Immune checkpoint, Immunotherapy

## Abstract

**Purpose:**

Medulloblastomas (MB) are highly malignant brain tumors that predominantly occur in young infants. Immunotherapy to boost the immune system is emerging as a novel promising approach, but is often hampered by inhibitory immune checkpoints. In the present study, we have studied immune checkpoint B7-H3 expression in a tissue cohort of human pediatric MB.

**Methods:**

Expression of B7-H3 was detected by immunohistochemistry and classified via B7-H3 staining intensity and percentage of B7-H3 positive tumor cells. Subsequently, B7-H3 protein expression was distinguished in MB molecular subtypes and correlated to immune cell infiltrates, patient characteristics, and survival.

**Results:**

B7-H3 protein expression was found in 23 out of 24 (96%) human pediatric MB cases and in 17 out of 24 (71%) MB cases > 25% of tumor cells had any level of B7-H3 expression. B7-H3 protein expression was more frequent on Group-4 MB as compared with other molecular subtypes (*p* = 0.02). Tumors with high B7-H3 expression showed less influx of γδT cells (*p* = 0.002) and CD3+ T cells (*p* = 0.041).

**Conclusion:**

Immune checkpoint B7-H3 is differentially expressed by the large majority of pediatric MB. This further warrants the development of novel B7-H3-directed (immuno)therapeutic methods for children with incurable, metastatic, or chemo-resistant MB.

## Introduction

Medulloblastoma (MB) is the most common malignant brain tumor that typically occurs during childhood, accounting for 20–25% of all pediatric brain tumors world-wide [1]. Clinical treatment of MB is limited to neurosurgical removal of the tumor and radiation therapy, together with chemotherapy [2]. Relatively late diagnosis, metastasis, long-term side effects, and high rate of relapses are factors that contribute to low survival chances of MB patients [2]. In general, defined molecular subgroups of MB are wingless (WNT, about 10% of MB cases), sonic hedgehog (SHH, about 30% of MB cases), group 3 (about 25% of cases), and group 4 (about 35% of cases) [2]. Prognostic outcome of MB patients depend on the molecular features of these subgroups, with extremely favorable prognosis for WNT tumors, intermediate outcomes for SHH and group 4 tumors, and poor prognosis for group 3-driven MB [2].

Immunotherapy with immune checkpoint inhibitors to enhance the immune response is emerging as a promising novel therapy to improve survival and to avoid relapses in brain cancer. For instance, blocking PD1-PD-L1 immune checkpoints with antibodies (e.g. nivolumab and pembrolizumab) result in improved therapy response in non-small cell lung cancer (NSCLC), melanoma, and their corresponding brain metastases [3, 4]. PD-L1 is also expressed by several brain cancers, including glioblastoma, and clinical trials are ongoing. However, PD-L1 expression by MB is limited if not absent [5], indicating that interfering with the PD-1/PD-L1 axis may not be feasible in MB. The expression landscape of immune checkpoints on MB remains unclear. Recently, immune checkpoint B7-H3 (CD276) has been detected on the plasma membrane of MB cells [6–9]. As such, B7-H3 might be an interesting therapeutic target for blocking antibodies or for chimeric antigen receptor (CAR) T cell therapy [7]. However, the evidence of B7-H3 expression by MB is based on limited cases, cell lines instead of tissue, non-validated antibodies for B7-H3 detection [6, 8], single scoring systematics, lack of correlation with molecular tumor subtype, clinical data or microenvironment, or via analyses on 0.6–1 mm cancer tissue cores in a tissue microarray [7, 10]

In the present study, we determined immune checkpoint B7-H3 protein expression in MB, using a knockout-validated anti-B7-H3 antibody in immunohistochemistry on whole tissue slides of 24 pediatric MB cases. Furthermore, we have distinguished B7-H3 protein expression in MB molecular subtypes and correlated these to immune cell infiltrates, patient characteristics, and survival.

## Materials and methods

### Patients

Our cohort of pediatric MB has been described by Vermeulen et al. [11]. The study material was derived from the archive of the Department of Pathology of the University Medical Center Utrecht, Utrecht, The Netherlands and distributed by the Biobank of the Department of Pathology that is overseen by the institutional medical ethical review board. Since we use archival pathology material which does not interfere with patient care and does not involve physical involvement of the patient, no ethical approval is required according to Dutch legislation. Use and storage of anonymous or coded left over material for scientific purposes is part of the standard treatment contract with patients and therefore informed consent procedure was not required according to our institutional medical ethical review board.

### Immunohistochemistry

Immunohistochemistry was carried out on 4 μm thick formalin-fixed, paraffin-embedded sections as we described [12]. Briefly, endogenous peroxidase activity was blocked for 15 min in a buffer containing 0.3% hydrogen peroxide. After antigen retrieval, i.e. boiling for 20 min in 10 mM citrate pH6.0, or Tris/EDTA pH9.0. After boiling for 20 min in 10 mM citrate pH6.0, staining was performed with B7-H3(CD276) antibody (NBP1-88966, Novus Biologicals, Littleton, USA; knockout-validated; 1:70) in PBS containing 2% BSA for 1 h at room temperature. The signal was amplified using Bright vision poly-HRP anti-rabbit (DPVO-HRP, Immunologic) and developed with diaminobenzidine followed by counterstaining with haematoxylin, dehydration in alcohol and mounting. Appropriate positive and negative controls were included in all stainings.

### Scoring of immunohistochemistry

Scoring and grading of immunohistochemistry was executed blindly by three independent observers, using the scoring table by Gregorio et al. [6]. The immunohistochemical results were classified via two different ways. One method was qualitatively scored as 0 in the absence of reactivity or < 10% of cells with membranous staining; score 1 in the presence of weak and partial membranous reactivity in > 10% of cells; score 2 when moderate membranous reactivity was detected in > 10% of cells; and score 3 when intense membranous reactivity occurred in > 10% of cells. Using another scoring method, reactivity was graded semi-quantitatively as: ± with 0–25% positive tumor cells; + with 25–50% positive tumor cells; ++ with 50–75% positive tumor cells; and +++ with 75–100% positive tumor cells.

### Statistical analysis

Statistical analysis was performed using IBM SPSS version 23 (SPSS Inc.). A Median Split was used for turning a continuous variable into a categorical one. Any value below the median is put it the category ‘Low’ and any value above median labeled ‘High’. Categorical data were analyzed using the Fisher’s exact test (for gender and molecular classification). Immune cell infiltrates was analyzed using Pearson chi-squared test. Overall survival was used as outcome endpoint and defined as the time between date of surgery and death. Censored patients were confirmed alive at time of censoring. Survival data were analyzed using the Kaplan–Meier method and log-rank test, taking all patients into account. Statistical significance was set at *p* < 0.05.

## Results

**Fig. 1:**
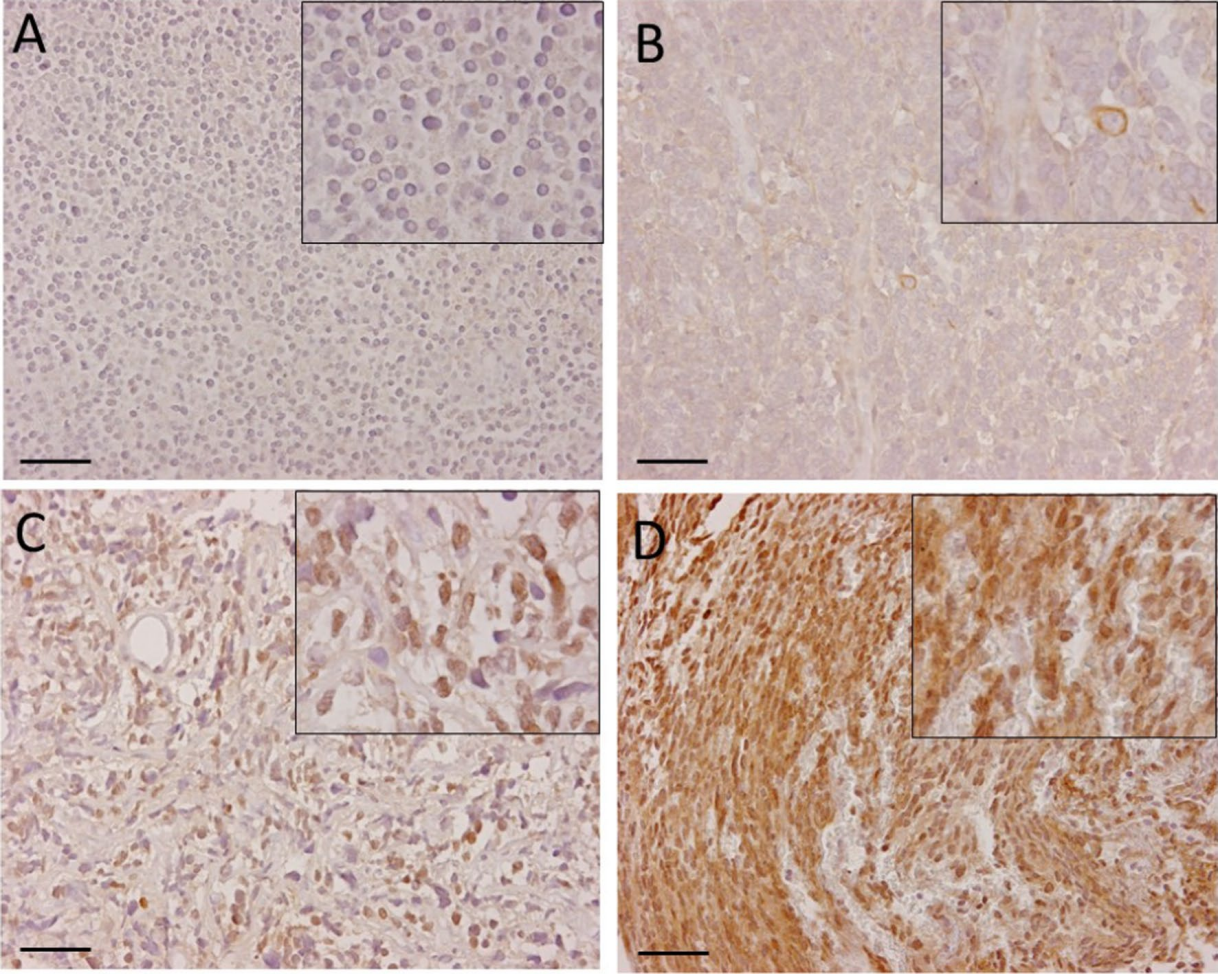
Expression of B7-H3 in pediatric medulloblastoma. **A** B7-H3 expression scored with score 0 and grade ±. **B** B7-H3 expression scored with score 1 and grade +. **C** B7-H3 expression scored with score 2 and grade ++. **D** B7-H3 expression scored with score 3 and grade +++. Right corner corresponding to the inset. Original magnification × 20, insets × 40, scale bar 50 µm

**Fig. 2:**
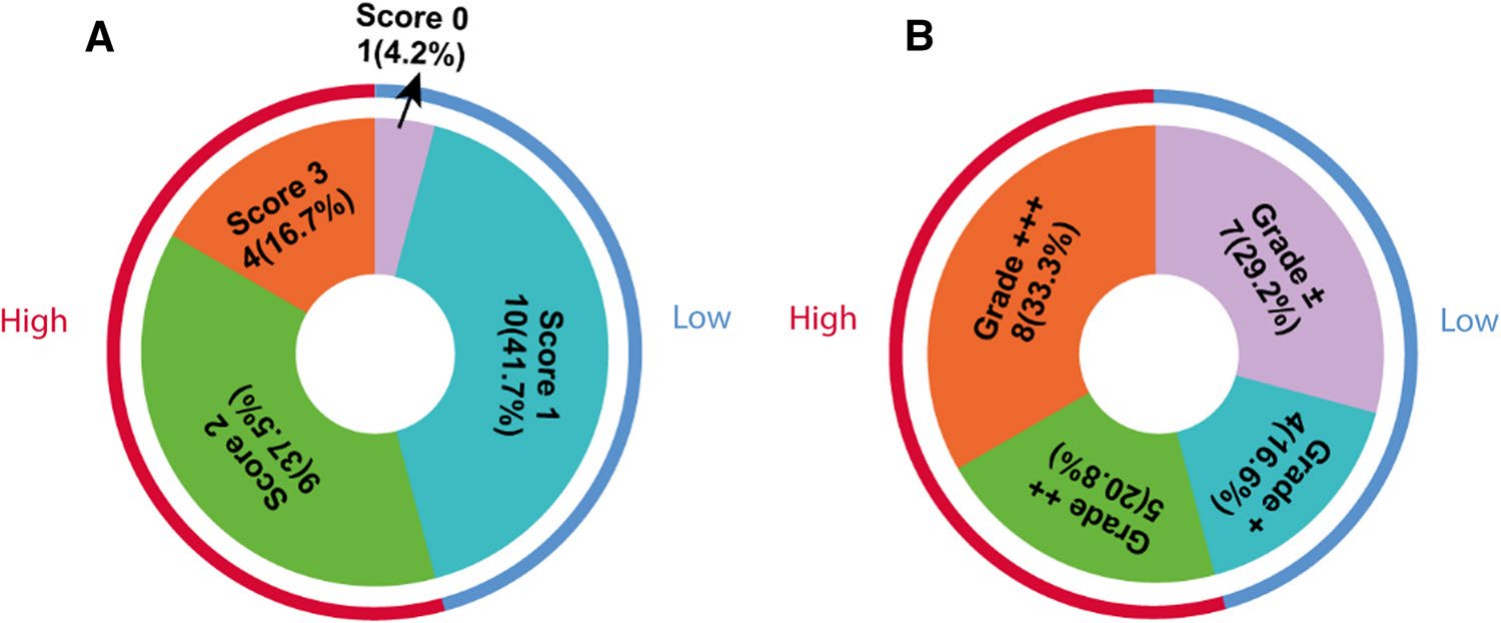
Classification of medulloblastoma. **A** Classification of different intensity scores (0, 1, 2, 3) for B7-H3. Score 2 and score 3 defined as high expression of B7-H3 (*n* = 13, outside red line), score 0 and score 1 defined as low expression of B7-H3 (*n* = 11, outside blue line). **B** Classification of B7-H3 positive staining in tumor cells. Grade ++ and grade +++ defined as high expression of B7-H3 (*n* = 13, outside red line), grade ± and grade + defined as low expression of B7-H3 (*n* = 11, outside blue line)

We investigated B7-H3 expression in a cohort of human MB tissues (Table 1). B7-H3 expression was found in 23 out of 24 MB cases (96%) and in 17 out of 24 (71%) tumors > 25% of tumor cells had any level of B7-H3 expression. Representative immunohistochemical stainings of B7-H3 are depicted in Fig. 1. First, tumors were scored for B7-H3 staining intensity and separated in four categories: (1) no membranous staining or any staining in < 10% of cells (*n* = 1), (ii) weak and partial membranous staining (*n* = 10), (iii) moderate membranous staining (*n* = 9), and (4) intense membranous staining (*n* = 4) (Fig. 2A). Second, we determined the percentage of B7-H3 positive tumor cells: < 25% (*n* = 7), 25–50% (*n* = 4), 50–75% (*n* = 5), and > 75% (*n* = 8) (Fig. 2B). B7-H3 protein expression was more frequent on group-4 MB as compared with other molecular subtypes (*p* = 0.02). No correlation was found between B7-H3 protein expression (reactivity score) and gender (*p* = 0.300), molecular subtypes (*p* = 0.077), or overall survival (*p* = 0.181). Previously, we have measured immune cell infiltrates in these MB tumor tissues, including CD3+ T cells, CD4+ T cells, CD8+ T cells, CD20+ B cells, NK cells, FOXP3+ regulatory T cells, and γδT cells [11]. We found that tumors with a high B7-H3 score (2 or 3) showed less influx of γδT cells (*p* = 0.002) and CD3+ T cells (*p* = 0.041), whereas the influx of CD4- or CD8-positive T cells, B cells or NK cells was not correlated to B7-H3 expression (Table 2). In conclusion, we have demonstrated that immune checkpoint B7-H3 is differentially expressed by the large majority of pediatric MB.

**Table 1:** Patient characteristics

	*N* or value	%
Gender		
Male	15	62.5
Female	9	37.5
Age (years)		
Mean ± SD	8.6 ± 5.2	
Range	0.4–17.8	
Histological type		
Classic	14	58.3
Desmoplastic nodular	6	25.0
Extensive nodular	3	12.5
Anaplastic	1	4.2
Molecular classification		
WNT	1	4.2
SHH	8	33.3
Group 3	5	20.8
Group 4	8	33.3
Undetermined	2	8.3

**Table 2:** Correlation between B7-H3 and infiltrating immune cells

Infiltrating immune cells	B7-H3 score				*p* values^a^
	Low	Ratio (%)	High	Ratio (%)	
CD3+					
Low infiltrating	3	25	9	75	0.041*
High infiltrating	8	66.7	4	33.3	
CD4+					
Low infiltrating	3	27.3	8	72.7	0.093
High infiltrating	8	61.5	5	38.5	
CD8+					
Low infiltrating	4	33.3	8	66.7	0.219
High infiltrating	7	58.3	5	41.7	
CD20+					
Low infiltrating	4	33.3	8	66.7	0.219
High infiltrating	7	58.3	5	41.7	
NKp46+					
Low infiltrating	5	35.7	9	64.3	0.239
High infiltrating	6	60	4	40	
FOXP3+					
Low infiltrating	4	33.3	8	66.7	0.219
High infiltrating	7	58.3	5	41.7	
γδTCR+					
Low infiltrating	1	10	9	90	0.002**
High infiltrating	8	80	2	20	

Numbers and percentage of medulloblastoma patients were showed in different subgroups

^a^A Pearson Chi-square analysis was used to evaluate correlation between B7-H3 expression (score of intensity staining) and infiltrating immune cells. Score 2 and score 3 defined as high expression of B7-H3 (*n* = 13), score 0 and score 1 defined as low expression of B7-H3 (*n* = 11)

*p < 0.05, **p < 0.01

## Discussion

We showed that immune checkpoint B7-H3 is differentially expressed by the large majority of pediatric MB. It would have been of interest to determine whether B7-H3 expression could be used as an independent prognostic marker. However, the amount of patient cases available are too low to execute proper multi-variate statistical analyses. Nevertheless, our data further warrant the development of novel immunotherapeutic methods for children with incurable, metastatic, or chemo-resistant MB. First, B7-H3 may serve as a novel immune checkpoint inhibitor target for MB. To date, little is known about the molecular interactions, receptor(s), and biological functions of B7-H3 in cancer. However, B7-H3-deficient mice, or mice treated with an antagonistic antibody to B7-H3, showed reduced growth of many tumors, including lymphoma and lymphoblast [13]. Deficiency or blocking B7-H3 leads to increased CD8+ T cell and NK cell function in tumor-bearing mice [13]. Fukushima et al. reported that B7-H3 negatively regulated both Th1- and Th2-mediated immune responses [14]. This is consistent with our finding of low CD3+ T cell influxes when B7-H3 expression scores are high. Clinical trials with B7-H3 blocking antibody 8H9, also in combination with PD-1 blockade, are currently ongoing for treating patients with recurrent metastatic neuroblastoma and desmoplastic small round cell tumors in the peritoneum [15]. The wide expression of B7-H3 on MB appeals to the exploration of immunotherapeutic targeting B7-H3 in MB as well. Second, CAR T cell therapy is an upcoming approach designed to arm cytotoxic T lymphocytes with a receptor that can recognize a surface protein on tumor cells [16]. For instance, CD19-directed CAR T cells have previously been successful in treating certain pediatric hematologic malignancies [16]. Likewise, B7-H3-directed CAR T cells can mediate significant regression of MB tumors in xenograft mouse models [7]. In the current study, we confirmed that checkpoint B7-H3 is differentially expressed by the large majority of pediatric MB, using whole tissue slides, and we related this for the first time to MB molecular subtype and immune cell infiltrates. Thus, B7-H3 CAR T cells could represent an interesting novel therapeutic option for children with incurable, metastatic, or chemo-resistant MB.
